# Heart beats: not to be beaten

**DOI:** 10.1007/s12471-015-0725-2

**Published:** 2015-07-02

**Authors:** E.E. van der Wall

**Affiliations:** Netherlands Society of Cardiology/Holland Heart House, Moreelsepark 1, 3511 EP Utrecht, The Netherlands

The summer 2015 issue of the *Netherlands Heart Journal* (NHJ) deviates from the regular NHJ issues. Following the usual Editor’s Comment [1], the July/August 2015 issue starts with a review article on cardiovascular genetics by Asselbergs et al. [2]. In this review, the genetic variants, mutations and inheritance models are briefly introduced, after which an overview is provided of current clinical and technological applications in gene diagnostics and research for cardiovascular disease and in particular, dilated cardiomyopathy. Following this very elucidating review article, the present issue is almost entirely dedicated to Heart Beats, a category that was initiated last year in our journal to replace the Case Reports and Images [3]. Heart Beats offer more concise and succinct information than case reports and do not need to include images per se.

With respect to the current issue, we have selected 12 Heart Beats on five different topics: atherothrombosis, electrophysiology, congenital heart disease, cardiac surgery and cardiovascular imaging. Within the topic of atherothrombosis, Delsing et al. [4] describe the diagnostic dilemmas in a patient with multivascular embolic stroke who turned out to have pancreatic cancer. The thrombotic complications of pancreatic cancer, in combination with a large, patent foramen ovale, support the mechanism of a paradoxical embolism through the patent foramen ovale as the cause of cerebral ischaemic stroke. Arslan et al. [5] describe acute pontine infarction after percutaneous coronary intervention: a very rare but devastating complication. It is the first report of a brain stem infarction as a complication of percutaneous coronary intervention. Within the electrophysiology theme, De Roeck et al. [6] discuss retrograde access of the left atrium for pulmonary vein isolation using magnetic navigation after closure of an atrial septal defect. This approach proved to provide a feasible, safe and successful alternative for catheter ablation of cardiac arrhythmias in patients in whom the classic trans-septal approach is impossible. Ramdjan et al. [7] report successful endovascular catheter ablation of ventricular tachycardia in a patient with a surgically repaired congenital left ventricular aneurysm. Within the topic of electrophysiology, we have also published two Letters-to-the Editor in a response-and-answer format. Kumar et al. [8] comment on the first Dutch experience with the second-generation endoscopic laser balloon ablation system for the atrial fibrillation published by Gal et al. [9] in a previous issue of NHJ. This comment was responded by Gal and Elvan [10].

Within the subject of congenital heart disease, Alsemgeest et al. [11] describe the first patient with a rapidly progressive dilated cardiomyopathy caused by an atypical aortic coarctation with a rapid normalisation of ventricular function after treatment by stenting. Pans et al. [12] evaluate the haemodynamics in a patient with Fontan physiology undergoing laparoscopic cholecystectomy. Laparoscopic surgery showed advantages over open surgery: less postoperative pain, shorter hospital stay, a reduction in postoperative wound infections and a reduction of respiratory complications.

Within the topic of cardiac surgery, van Houte et al. [13] report on the use of percutaneous femoral veno-arterial extracorporeal membrane oxygenation (VA-ECMO) in a fully awake, non-intubated and spontaneously breathing patient suffering from acute, severe and refractory cardiogenic shock due to a (sub)acute anterior myocardial infarction. This limited invasive approach may encourage a more widespread use of percutaneous VA-ECMO. Gerds et al. [14] describe a patient with left ventricular assist device thrombosis who has increased lactate dehydrogenase levels, associated with clinical deterioration and subsequent right heart catheterisation. It was suggested that only pump exchange is life-saving in those patients. Halim et al. [15] assess the relationship between the formation of a giant intra-cardiac blood cyst and previous cardiac surgery. Their report shows that the presence of an acquired intra-cardiac cyst is a rare entity and that a pathophysiological association between the formation of a blood cyst and previous cardiac surgery has not been proven yet.

Finally, within the cardiovascular imaging theme, Engbers et al. [16] report zero coronary calcium in the presence of three-vessel and left main coronary artery disease in a Hodgkin’s lymphoma survivor. As a result, a calcium score of zero does not rule out severe obstructive coronary artery disease. Whether mediastinal radiation therapy is associated with coronary artery disease without accompanying calcium deposits remains unclear. Adriaans et al. [17] describe a patient with progressive dyspnoea using non-invasive imaging and autopsy findings. The article showed the strong ability of non-invasive diagnostic techniques to detect cardiac involvement in advanced systemic amyloid light-chain amyloidosis, which was confirmed at autopsy. Baur et al. [18] used advanced imaging techniques to visualise a patient with a phaeochromocytoma. The patient had several cardiovascular complications, such as rapid atrial arrhythmias and poor left ventricular function, to be attributed to the tumour. After removal of the tumour, cardiac function returned to normal.

To summarise, the present issue of NHJ reports 12 unique clinical cases from five different cardiovascular areas. It is hoped that the readers will appreciate the general clinical impact of the individual findings.
